# Dr. Horace C. Taylor

**Published:** 1904-01

**Authors:** 


Dr. Horace C. Taylor, of Brocton, N. Y., died at his home, December 21, 1903, aged 90 years. He was among the oldest and best-known physicians of Chautauqua County.

